# Expression of cancer-associated fibroblast related proteins in metastatic breast cancer: an immunohistochemical analysis

**DOI:** 10.1186/s12967-015-0587-9

**Published:** 2015-07-11

**Authors:** Hye Min Kim, Woo Hee Jung, Ja Seung Koo

**Affiliations:** Department of Pathology, Severance Hospital, Yonsei University College of Medicine, 50 Yonsei-ro, Seodaemun-gu, Seoul, 120-752 South Korea

**Keywords:** Breast cancer, Cancer-associated fibroblast, Molecular subtype, Stroma

## Abstract

**Background:**

Cancer-associated fibroblast (CAF) is the most studied element of the tumor microenvironment, although no relationship has been identified between expression of their related proteins and the metastasis site. The purpose of this study was to investigate the expression of CAF related proteins and their implications according to the metastasis site in metastatic breast cancer.

**Methods:**

Immunohistochemical staining was used to evaluate the expression of CAF related proteins (podoplanin, prolyl 4-hydroxylase, FAPα, S100A4, PDGFRα, PDGFRβ, and NG2) in tissue microarrays from 132 cases of metastatic breast cancer (bone metastasis: 32 cases, brain metastasis: 38 cases, liver metastasis: 10 cases, and lung metastasis: 52 cases). Breast cancer subtypes were classified as luminal A, luminal B, HER-2, and triple negative breast cancer, according to the immunohistochemical staining results for estrogen and progesterone receptors, HER-2, and Ki-67 and FISH results for HER-2. Tumors were classified as desmoplastic, sclerotic, normal-like, and inflammatory type, according to the histologic findings from the tumor stroma.

**Results:**

Various CAF related protein expression profiles were observed, according to the metastasis site. For bone metastasis, the expression of stromal podoplanin, S100A4, and PDGFRα was significantly high. For lung metastasis, the expression of stromal PDGFRβ was significantly elevated (p < 0.001). For liver metastasis, significantly reduced expression of stromal S100A4 (p = 0.002) and PDGFRα (p = 0.011) was observed. Expression of CAF related proteins also differed according to the stromal phenotype. Desmoplastic stroma exhibited significantly elevated expression of stromal podoplanin (p < 0.001), S100A4 (p < 0.001), PDGFRα (p = 0.010), and PDGFRβ (p = 0.021). Inflammatory stroma exhibited significantly elevated expression of stromal FAPα (p = 0.044) and significantly reduced stromal S100A4 expression (p < 0.001). Sclerotic stroma exhibited significantly elevated tumoral FAPα (p = 0.005) expression. For lung metastasis, shorter overall survival was significantly related to tumoral podoplanin expression (p = 0.006), stromal podoplanin expression (p = 0.018), tumoral prolyl 4-hydroxylase negativity (p = 0.016), and tumoral PDGFRα expression (p = 0.001).

**Conclusion:**

For metastatic breast cancer, significant differences were observed in the expression of CAF related proteins, according to the metastasis site and stromal histologic phenotype.

**Electronic supplementary material:**

The online version of this article (doi:10.1186/s12967-015-0587-9) contains supplementary material, which is available to authorized users.

## Background

Breast cancers are associated with high morbidity and mortality rates, as they are prone to distant metastasis. The major metastasis sites for breast cancer are the lungs, brain, liver, and bone [1, 2], and most studies have been evaluated in brain and bone metastasis [3–8]. The most common mechanism for tumor metastasis is a reciprocal interaction between the tumor cell and host tissue, which is achieved by adhesion, proteolysis, invasion, and angiogenesis [2, 9]. In addition, the importance of the tumor microenvironment in the process of metastasis has recently emerged. Among the various elements of the tumor microenvironment, cancer-associated fibroblast (CAF) is the most studied, and are currently considered most important element in this microenvironment [10]. Various proteins have been suggested as markers for CAF, including α-SMA [11], tenascin-C [12], chondroitin sulfate proteoglycan (NG2) [13], platelet-derived growth factor receptors (PDGFR)α/β [14], fibroblast activation protein (FAP) [15], podoplanin [16], prolyl 4-hydroxylase [17], and fibroblast-specific protein (FSP)-1/S100A4 [13]. Therefore, it appears that CAF may consist of various functional subtypes. In one recent study, CAF were classified into various subsets, such as FAPα type, FSP1 type, PDGFRα type, and PDGFRβ type [18]. These subsets all showed different characteristics; this supports the hypothesis that CAF are comprised of diverse phenotypes.

To explain the unique metastasis pattern for each cancer, the “seed and soil” hypothesis has been proposed, which explains how a specific tumor (the seed) survives in a specific visceral organ (the soil) [19]. Similarly, different characteristic findings have been reported according to the site of metastasis for metastatic breast cancer. For example, previous studies have demonstrated that brain metastasis was related to young age, estrogen-receptor (ER) negativity, prior lung metastasis, HER-2 overexpression, epidermal growth factor receptor (EGFR) overexpression, and the basal subtype [5–7]. In addition, bone metastasis was related to a lower histologic grade, ER positivity, ER positivity and progesterone receptor (PR) negativity, strand growth patterns, and the presence of fibrotic foci in invasive ductal carcinoma [4, 20, 21]. Thus, as the characteristics of metastatic breast cancer differed according to the site of metastasis, it is likely that there are similar phenotypic differences for CAF, although few studies have evaluated this topic. Therefore, the purpose of this study was to investigate the expression of CAF related proteins and their implications, according to the metastasis site in metastatic breast cancer.

## Methods

### Patient selection

This study retrospectively reviewed cases of invasive primary breast cancer with metastasis to distant organs (liver, lungs, brain, and bone) from the records of the Department of Pathology, Severance Hospital, South Korea. Only patients that were diagnosed with invasive ductal carcinoma were included, which provided a total of 132 cases, including 49 cases with paired primary and metastasized tumors. All slides from these cases were reviewed, and the pathologic parameters were evaluated by 2 pathologists (JSK and WHJ). The histologic grade was assessed using the Nottingham grading system [22]. The study design was reviewed and approved by our Institutional Review Board.

For the invasive ductal carcinomas, the tumor stroma was classified according to the microscopic findings as desmoplastic type (stroma consisting of cellular fibroblast/myofibroblast proliferation), sclerotic type (stroma with fibrotic collagenous components and minimal cellular components), pauci-stromal type (minimal stromal tissue near the tumor), or inflammatory type (stroma consisting of inflammatory cells, such as lymphocytes).

### Tissue microarrays

On H&E-stained slides of tumors, a representative area was selected, and a corresponding spot was marked on the surface of a paraffin block. Using a biopsy needle, the selected area was extracted, and a 3-mm tissue core was placed into a 6 × 5 array. Tissues from the invasive tumor were extracted, and more than two tissue cores were extracted to minimize extraction bias. Each tissue core was assigned a unique tissue microarray location number, which was linked to a database that contained the other clinicopathological data.

### Immunohistochemistry

The antibodies and dilutions that were used for the immunohistochemistry are listed in Additional file 1: Table S1. All immunohistochemistry was performed using the formalin-fixed and paraffin-embedded tissue sections. Briefly, 5-μm sections were obtained using a microtome, transferred onto adhesive slides, and dried at 62°C for 30 min. After incubation with the primary antibodies, immunodetection was performed using biotinylated anti-mouse immunoglobulin, followed by peroxidase-labeled streptavidin using a labeled streptavidin biotin kit with 3,3′-diaminobenzidine chromogen as the substrate. The primary antibody incubation step was omitted in the negative control. Positive control tissue was used as per the manufacturer’s recommendation. The slides were subsequently counterstained with Harris hematoxylin.

### Interpretation of immunohistochemical staining

All immunohistochemical markers were assessed via light microscopy. A cut-off value of ≥1% positively stained nuclei was used to define ER and PR positivity [23]. HER-2 staining was analyzed according to the American Society of Clinical Oncology/College of American Pathologists guidelines, using the following categories: 0 = no immunostaining; 1+ = weak incomplete membranous staining in <10% of the tumor cells; 2+ = complete membranous staining, either uniform or weak, in ≥10% of the tumor cells; and 3+ = uniform intense membranous staining in ≥30% of the tumor cells [24]. For our analysis, HER-2 immunostaining was considered positive when strong (3+) membranous staining was observed, whereas 0 or 1+ cases were considered negative. Cases with 2+ HER-2 expression were evaluated for HER-2 amplification via fluorescent in situ hybridization (FISH).

Immunohistochemical markers for podoplanin, prolyl 4-hydroxylase, FAPα, S100A4, PDGFRα, PDGFRβ, and NG2 were assessed via light microscopy. The stained slides were evaluated semi-quantitatively, as reported previously [25]. In brief, tumor and stromal cell staining was assessed as 0 = negative or weak immunostaining in <1% of the tumor/stroma, 1 = focal expression in 1–10% of the tumor/stroma, 2 = positive in 11–50% of the tumor/stroma, or 3 = positive in 51–100% of the tumor/stroma. These evaluations were performed for the entire tumor area, and scores of 2–3 were defined as positive for our analysis.

### Tumor phenotype classification

In this study, we classified the phenotypes of breast cancer according to the immunohistochemical results for ER, PR, HER-2, Ki-67, and FISH results for HER-2, as follows: luminal A type = ER and/or PR positive, HER-2 negative, and a Ki-67 labeling index (LI) of <14%; HER-2 negative luminal B type = ER and/or PR positive and Ki-67 LI ≥14%; HER-2 positive luminal B type = ER and/or PR positive, and HER-2 overexpressed and/or amplified; HER-2 overexpression type = ER and PR negative, and HER-2 overexpressed or/and amplified; and triple negative breast cancer (TNBC) type = negative for ER, PR, and HER-2 [26].

### Statistical analysis

Data were analyzed using SPSS for Windows (version 12.0, SPSS Inc., Chicago, IL, USA). Chi Square tests and Fisher’s exact tests were used for categorical variables, respectively. When analyzing data with multiple comparisons, we used the Bonferroni multiple comparison procedure to generate a corrected *p* value. Statistical significance was set at a p value of <0.05. Kaplan–Meier survival curves and log-rank statistics were used to evaluate the time to tumor recurrence and overall survival. Multivariate regression analysis was performed using the Cox proportional hazards model.

## Results

### Characteristics of the breast cancer cases

Among the 132 patients, 32 (24.2%) had bone metastasis, 38 (28.8%) had brain metastasis, 10 (7.6%) had liver metastasis, and 52 (39.4%) had lung metastasis. The proportion of patients with ER positivity and PR positivity was significantly elevated in bone and liver metastasis (p < 0.001), and the proportion of patients with HER-2 positivity was significantly elevated in brain metastasis (p = 0.047). A higher proportion of luminal A type was observed in bone and liver metastasis, and a higher proportion of TNBC type was observed in brain and lung metastasis (p < 0.001) (Additional file 1: Table S2). The immunohistochemical staining results and clinicopathologic characteristics of patients with metastatic breast cancer were determined in the metastatic tumor.

### Clinicopathological features according to the stromal phenotype

The stromal phenotype was analyzed, and 38 (28.8%) patients was classified as having desmoplastic stroma, 9 (6.8%) had inflammatory stroma, 45 (34.1%) had pauci-stroma, and 40 (30.3%) had sclerotic stroma. The clinicopathological characteristics were investigated according to the stromal phenotype and the only significant differences in stromal phenotype was according to the metastatic site (p < 0.001). The proportion of inflammatory and sclerotic stroma was higher in lung metastasis, desmoplastic stroma was more common in bone metastasis, and pauci-stroma was more common in brain metastasis (Table 1).

**Table 1:** Clinicopathological characteristics of the patients according to the breast cancer stromal histologic phenotype

Parameter	Total N = 132 (%)	Desmoplastic type n = 38 (%)	Inflammatory type n = 9 (%)	Pauci-stroma type n = 45 (%)	Sclerotic type n = 40 (%)	p value
Age (years)						0.600
≤50	68 (51.5)	21 (55.3)	5 (55.6)	25 (55.6)	17 (42.5)	
>50	64 (48.5)	17 (44.7)	4 (44.4)	20 (44.4)	23 (57.5)	
ER						0.339
Negative	63 (47.7)	20 (52.6)	6 (66.7)	22 (48.9)	15 (37.5)	
Positive	69 (52.3)	18 (47.4)	3 (33.3)	23 (51.1)	25 (62.5)	
PR						0.060
Negative	91 (68.9)	28 (73.7)	7 (77.8)	35 (77.8)	21 (52.5)	
Positive	41 (31.1)	10 (26.3)	2 (22.2)	10 (22.2)	19 (47.5)	
HER-2						0.370
Negative	89 (67.4)	28 (73.7)	4 (44.4)	29 (64.4)	28 (70.0)	
Positive	43 (32.6)	10 (26.3)	5 (55.6)	16 (35.6)	12 (30.0)	
Molecular subtypes						0.063
Luminal A	47 (35.6)	15 (39.5)	3 (33.3)	12 (26.7)	17 (42.5)	
Luminal B	23 (17.4)	3 (7.9)	0 (0.0)	11 (24.4)	9 (22.5)	
HER-2	27 (20.5)	7 (18.4)	5 (55.6)	8 (17.8)	7 (17.5)	
TNBC	35 (26.5)	13 (34.2)	1 (11.1)	14 (31.1)	7 (17.5)	
Ki-67 LI (%)						0.139
≤14	91 (68.9)	31 (81.6)	6 (66.7)	26 (57.8)	28 (70.0)	
>14	41 (31.1)	7 (18.4)	3 (33.3)	19 (42.2)	12 (30.0)	
Metastasis site						**<** *0.001*
Bone	32 (24.2)	18 (47.4)	0 (0.0)	4 (8.9)	10 (25.0)	
Brain	38 (28.8)	3 (7.9)	2 (22.2)	25 (55.6)	8 (20.0)	
Liver	10 (7.6)	0 (0.0)	0 (0.0)	0 (0.0)	10 (25.0)	
Lung	52 (39.4)	17 (44.7)	7 (77.8)	16 (35.6)	12 (30.0)	
Patients death	44 (33.3)	15 (39.5)	3 (33.3)	14 (31.1)	12 (30.0)	0.815

Italic value indicates statistically significant (p < 0.05).

*TNBC* triple negative breast cancer.

### Expression of CAF related proteins according to the metastasis site

**Figure 1:**
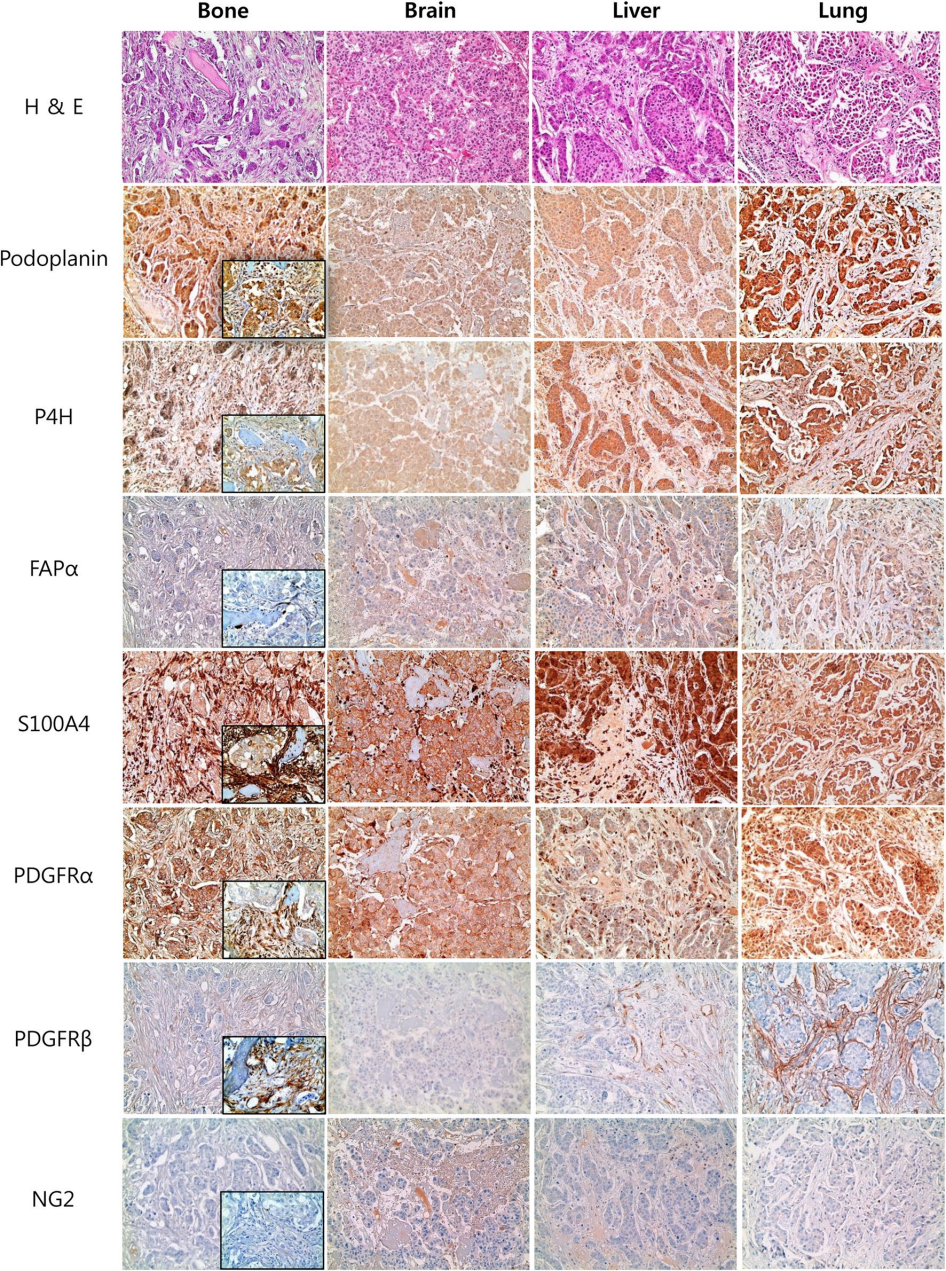
Expression of cancer-associated fibroblast related proteins according to the metastasis site. Stromal podoplanin, S100A4, and PDGFRα expression was elevated in bone metastasis, and stromal PDGFRβ expression was elevated in lung metastasis. In liver metastasis, stromal S100A4 and PDGFRα expression was reduced. *Inlet figures* show the expression of CAF related proteins in fibroblast.

**Figure 2:**
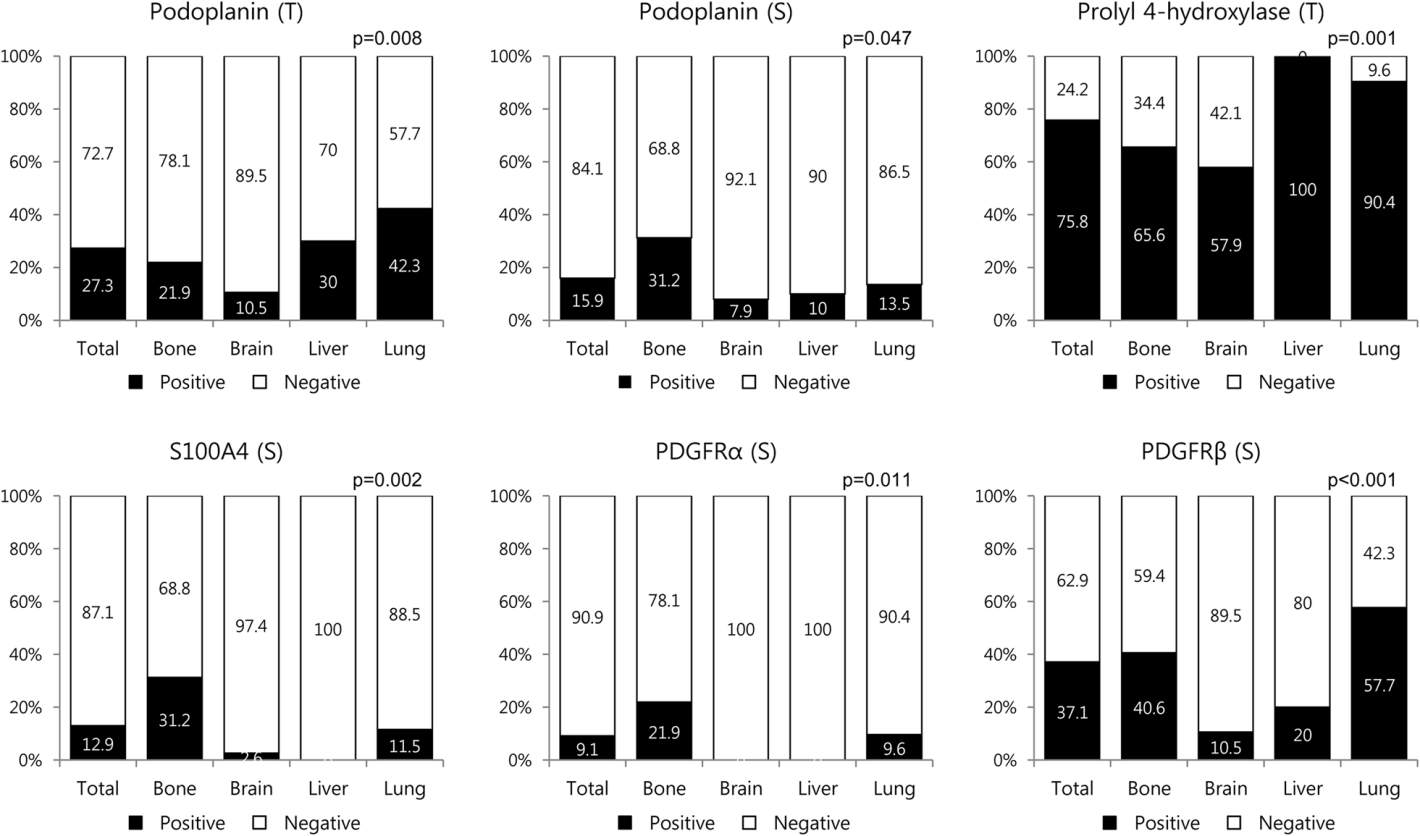
Expression of cancer-associated fibroblast related proteins in metastatic tumor according to the metastasis site. *T* tumor cell component, *S* stromal component.

Analysis of the expression of CAF related proteins in metastatic tumor according to the metastasis site revealed no expression of PDGFRβ and NG2 in the tumor cells, and no expression of prolyl 4-hydroxylase in the stromal component. However, significant differences were observed in the expression of tumoral podoplanin (p = 0.008), stromal podoplanin (p = 0.047), tumoral prolyl 4-hydroxylase (p = 0.001), stromal S100A4 (p = 0.002), stromal PDGFRα (p = 0.011), and stromal PDGFRβ (p < 0.001) according to the metastasis site. Stromal podoplanin, S100A4, and PDGFRα expression was elevated in bone metastasis, while tumoral podoplanin and stromal PDGFRβ expression was elevated in lung metastasis. In liver metastasis, tumoral prolyl 4-hydroxylase expression was elevated, while stromal S100A4 and PDGFRα expression was reduced. Expression of tumoral podoplanin, stromal podoplanin, tumoral prolyl 4-hydroxylase, stromal PDGFRα, and stromal PDGFRβ was reduced in brain metastasis (Figures 1, 2).

Analysis of the expression of CAF related proteins based on molecular subtype of each metastatic site revealed that stromal S100A4 (p = 0.015) and tumoral PDGFRα (p = 0.037) was associated with bone metastasis, tumoral prolyl 4-hydroxylase (p = 0.003) and tumoral S100A4 (p = 0.034) with brain metastasis, stromal podoplanin (p = 0.019) and tumoral S100A4 (p = 0.019) with liver metastasis, and tumoral PDGFRα (p = 0.034) and stromal PDGFRα (p = 0.042) with lung metastasis (Additional file 1: Table S3).

### Expression of CAF related proteins according to the stromal phenotype

**Figure 3:**
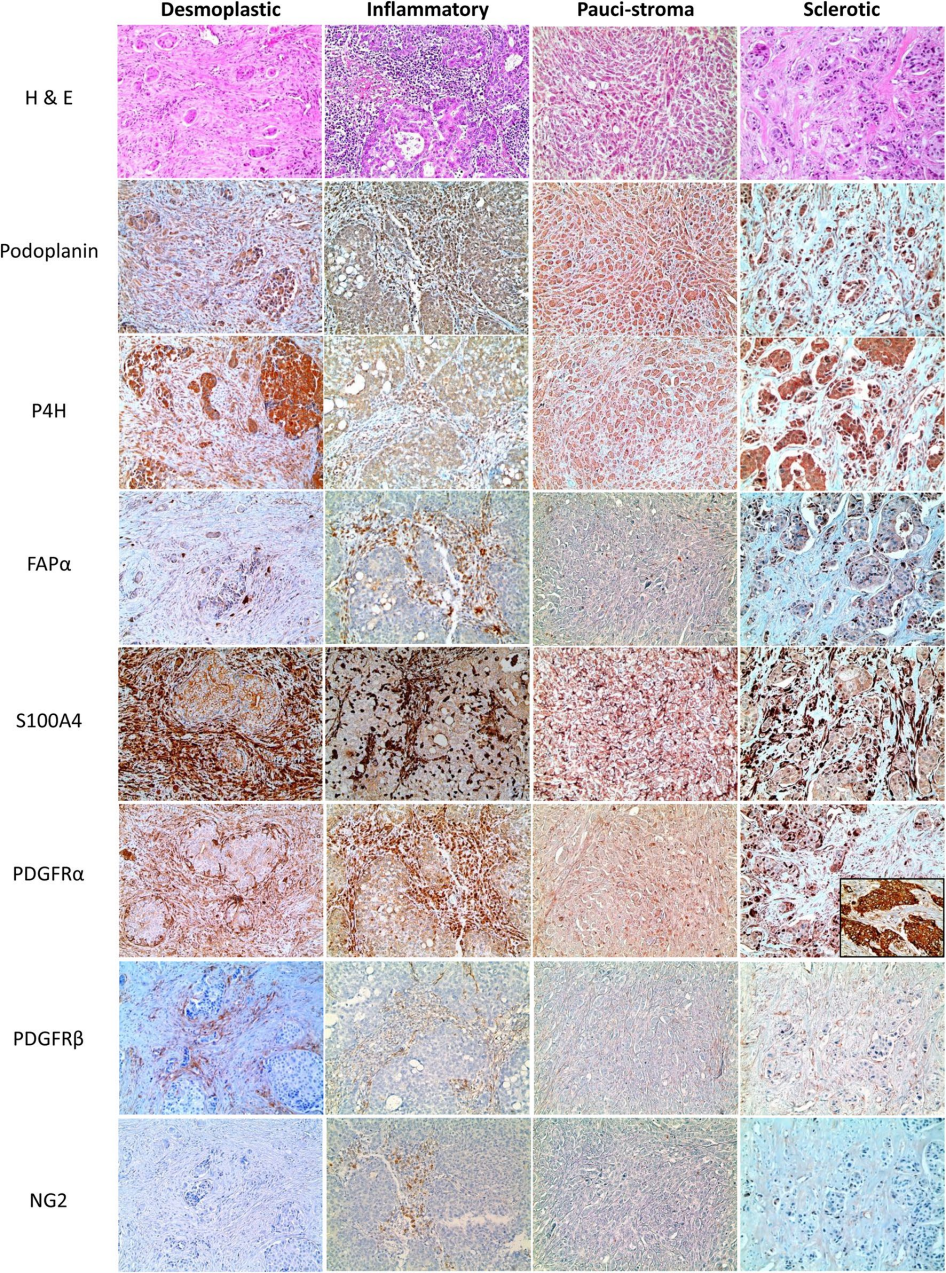
Expression of cancer-associated fibroblast related proteins according to the stromal phenotype. Desmoplastic stroma exhibited high expression of stromal podoplanin, S100A4, PDGFRα, and PDGFRβ, while inflammatory stroma exhibited high stromal FAPα expression. Sclerotic stroma exhibited high tumoral FAPα expression.* Inlet figures* show the expression of PDGFRα in cancer cells.

**Figure 4:**
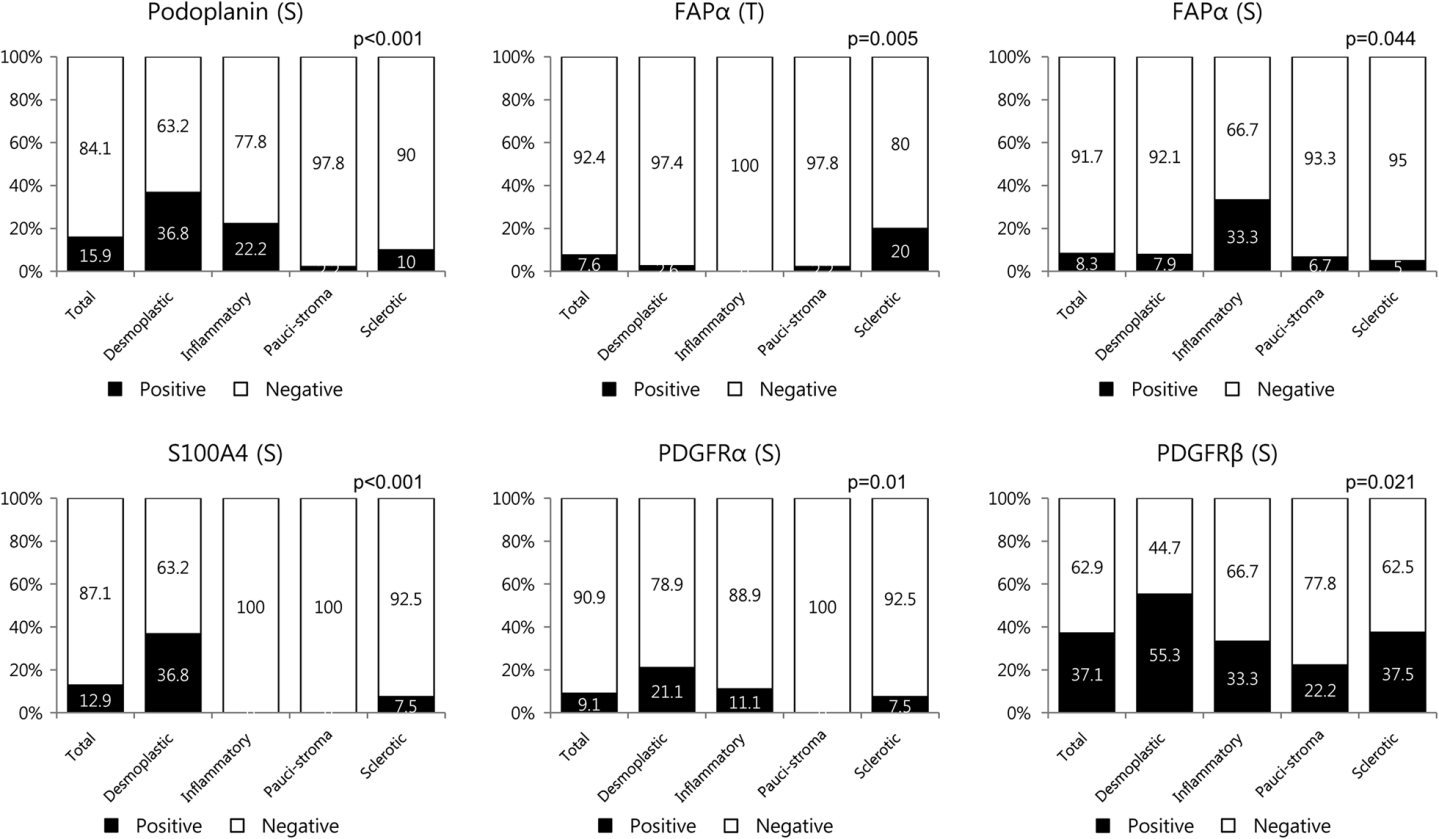
Expression of cancer-associated fibroblast related proteins in metastatic tumor according to the stromal phenotype. *T* tumor cell component, *S* stromal component.

We also investigated the expression of CAF related proteins according to the stromal phenotype, and observed significant differences in the expression of stromal podoplanin (p < 0.001), tumoral FAPα (p = 0.005), stromal FAPα (p = 0.044), stromal S100A4 (p < 0.001), stromal PDGFRα (p = 0.010), and stromal PDGFRβ (p = 0.021). Desmoplastic stroma exhibited high expression of stromal podoplanin, S100A4, PDGFRα, and PDGFRβ, while inflammatory stroma exhibited high stromal FAPα and low tumoral FAPα and stromal S100A4 expression. Sclerotic stroma exhibited low stromal FAPα and high tumoral FAPα expression, while pauci-stroma exhibited low expression of stromal podoplanin, S100A4, PDGFRα, and PDGFRβ (Figures 3, 4).

### Correlation of CAF related protein expression with primary and metastatic breast cancer

**Figure 5:**
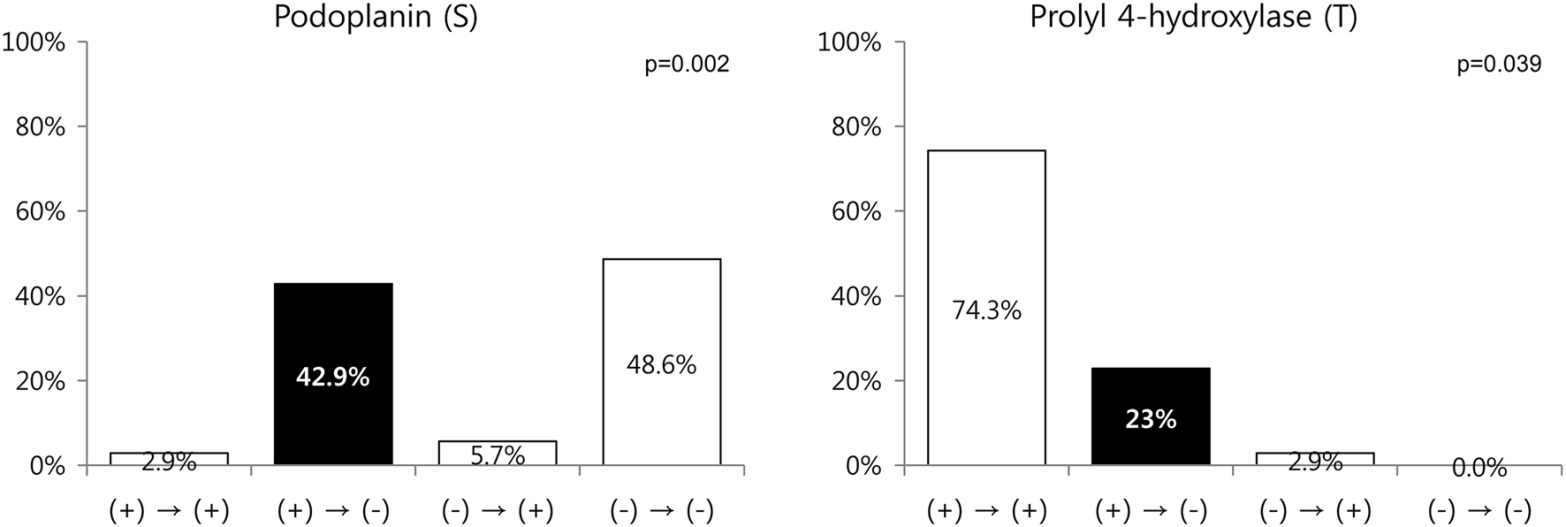
Correlation between cancer-associated fibroblast related protein expression for primary and metastatic cancer. Significant differences were observed in the expression of stromal podoplanin and tumoral prolyl 4-hydroxylase in primary and metastatic breast cancer.

Significant differences were observed in the expression of stromal podoplanin (p = 0.002) and tumoral prolyl 4-hydroxylase (p = 0.039) in primary and metastatic breast cancer. They were positive in primary cancer, but negatively converted in metastasis site in 42.9 and 22.9% cases, respectively (Figure 5). Each metastatic site-specific analysis did not show any significant findings; metastatic tumors other than lung metastasis were not included in the analysis, because the number is too small.

### Correlation between CAF related protein expression and the clinicopathological factors

**Figure 6:**
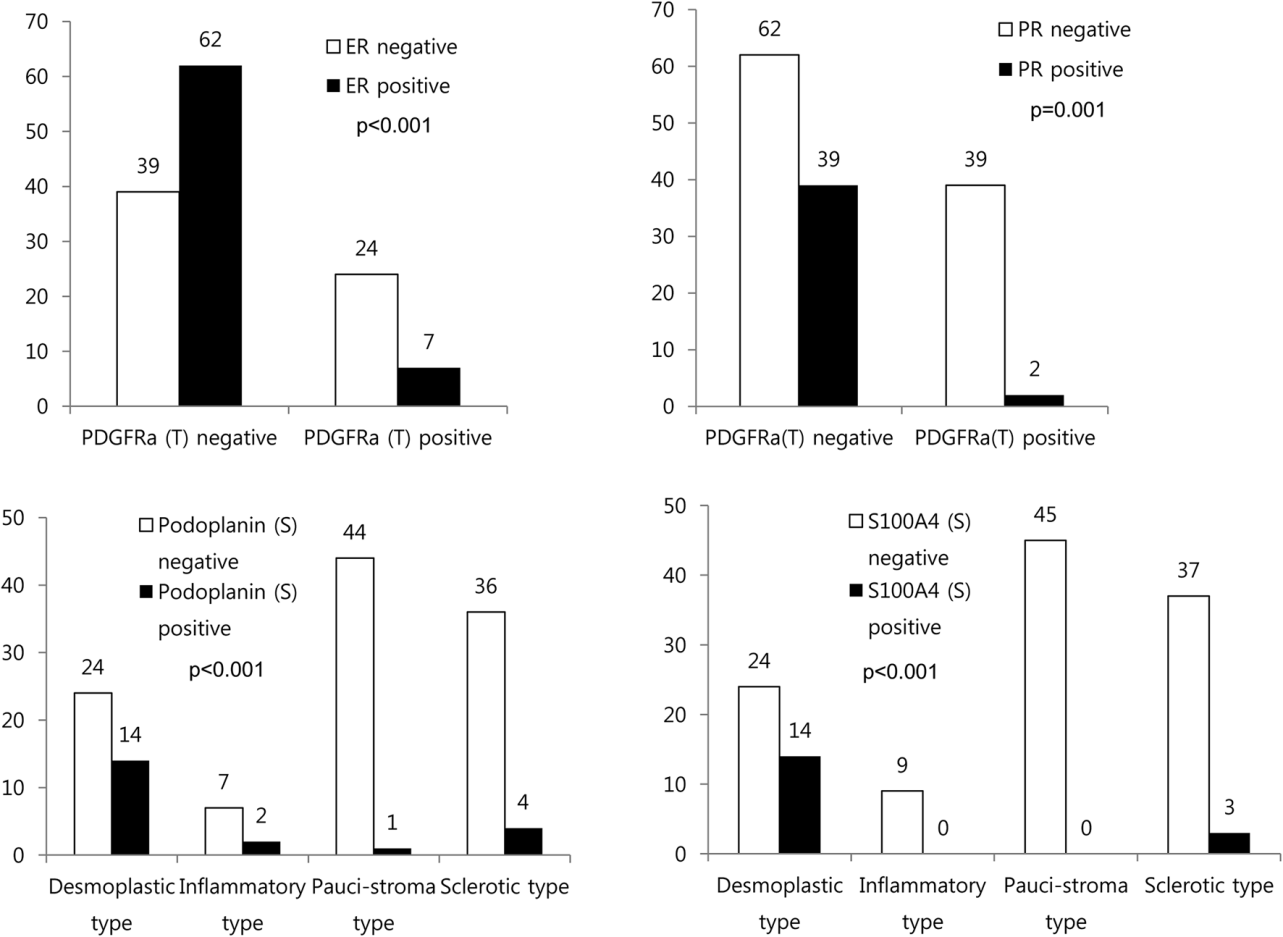
Correlation between the expressions of cancer-associated fibroblast related proteins and various clinicopathological factors.

The relationship between CAF related protein expression and the clinicopathological factors were investigated (Figure 6). Tumoral PDGFRα expression was associated with ER and PR negativity (p < 0.001 and p = 0.001, respectively). The stromal phenotype was associated with stromal podoplanin (p < 0.001) and S100A4 (p < 0.001) expression, with the desmoplastic stroma exhibiting high stromal podoplanin and S100A4 expression.

### Impact of CAF related protein expression on patient prognosis

When the impact of CAF related protein expression on patient prognosis was analyzed, no factor was associated with shorter overall survival (OS) in the univariate analysis (Additional file 1: Table S4).

## Discussion

Although no previous study has evaluated the expression of CAF related proteins according to the breast cancer metastasis site, the tumor stroma features are expected to be different, as different clinicopathological characteristics have been observed at different metastasis sites [27]. Therefore, we analyzed the expression of CAF related proteins according to the breast cancer metastasis site, and observed various different expression patterns. For example, the stromal expression of podoplanin, S100A4, and PDGFRα was high in bone metastasis, while PDGFRβ expression was high in lung metastasis. The high expression of podoplanin, S100A4, and PDGFRα in the CAF of bone metastasis is likely explained the characteristics of the bone environment. In a previous study, different functional characteristics were identified according to the CAF subtype [18], with PDGFRα and S100A4 types of CAF being related to macrophage infiltration and macrophage recruitment [28, 29]. It has also been suggested that bone is relevant to macrophage recruitment, because it is one of the main hematopoietic organs. In addition, a previous study has reported high expression of S100A4 in adipocyte-derived fibroblasts, which are a major desmoplastic stroma component in breast cancer [30]. As bone has numerous adipocytes in the marrow tissue, CAFs derived from marrow tissue are expected to exhibit high S100A4 expression. The second probable mechanism for our observed differences in CAF related protein expression is the differences in the metastatic cancer cell. For example, previous reports have reported metastasis site-specific characteristics in metastatic breast cancer, with bone metastasis exhibiting a lower histologic grade, ER positivity, ER positivity and PR negativity, strand growth patterns, and the presence of fibrotic foci in invasive ductal carcinoma [4, 20, 21]. As the tumor stroma characteristics are formed via reciprocal interactions with cancer cells, various CAF phenotypes would also be expected if the cancer cell characteristics varied according to the site of metastasis. In addition, we observed that the pathologic characteristics varied according to the metastasis site.

Therefore, different CAF profile might have influenced the tumor molecular subtype as well as different organ. However, our study indicates that CAF profile seems to be more related to different organ rather than tumor molecular subtype according to the following findings. First, there was no significant association between the expressions of CAF related proteins and molecular subtype by correlation analysis. Second, to evaluate the association between different organ and tumor molecular subtype in CAF profile, additional CAF profile by molecular subtype was evaluated according to metastatic site; if metastatic organ was identical, the items showing different expression of CAF related proteins by molecular subtype was fewer.

In lung metastasis, the CAF expression of PDGFRβ was high, and previous studies have reported a relationship between high interstitial fluid pressure and PDGFRβ type CAF [31]. Because the lungs have distinct histologic features, if a cancer cell metastasizes to a small interstitial tissue among the alveoli, it might encounter high interstitial fluid pressure, thereby promoting PDGFRβ expression. Furthermore, lung metastasis is known to be related to TNBC type; therefore, further investigation is needed to determine whether tumor cell characteristics might influence the CAF phenotype.

In this study, we demonstrated that CAF related protein expression varied according to the stromal histologic type, with desmoplastic stroma having high podoplanin, S100A4, PDGFRα, and PDGFRβ expression, and inflammatory stroma having high FAPα expression. Although a previous study classified breast cancer tumor stroma according to their histologic findings [32], few study has compared the differences in CAF according to the histologic findings, therefore these results are not comparable. In addition, it has been noted that CAF markers have specific and unique features. For example, in breast cancer, the desmoplastic response appears to be mediated by PDGF-AA signaling in PDGFRα type CAF [33]; this possibility is compatible with our findings. In addition, FAPα has been reported to possess an immunomodulatory function [18], which is consistent with our finding that inflammatory stroma has high FAPα expression. However, further investigation is needed to reveal the specific relationship between the tumor stroma histology and CAF characteristics.

The clinical implication of our findings is that CAF might be a potential anti-cancer therapeutic target. This target is particularly attractive, as it is genetically stable (relative to the cancer cell), exhibits distinct epigenetic changes within normal stromal cells, and can be targeted throughout the neoplasm process, as it supports and accompanies the cancer cell through the whole neoplasm spectrum [34]. Interestingly, several preclinical studies have targeted the CAF markers that we evaluated, and they reported that agents targeting CAF were effective in tumor inhibition [35–38]. However, to develop an effective cancer therapy, CAF-targeted treatments should exploit agents that target the specific CAF phenotype, and further studies are needed to determine which agents most effectively target each phenotype.

## Conclusion

In conclusion, the expression of CAF related proteins in stroma varies according to the breast cancer metastasis site and the stromal histologic phenotype.

## Additional file

Additional file 1:
**Table S1**. Source, clone, and dilution of the antibodies used. **Table S2**. Basal clinicopathologic characteristics of patients with metastatic breast cancer according to metastasis site. **Table S3**. Expression of CAF related proteins in the tumor cell compartment of metastatic breast cancer according to metastasis site.**Table S4**. Univariate analysis of the association between expression levels of CAF related proteins and overall survival by the log-rank test in metastatic breast cancer.

## Abbreviations

CAF: cancer-associated fibroblast
NG2: chondroitin sulfate proteoglycan
PDGFR: platelet-derived growth factor receptors
FAP: fibroblast activation protein
FSP: fibroblast-specific protein
ER: estrogen-receptor
EGFR: epidermal growth factor receptor
PR: progesterone receptor
FISH: fluorescent in situ hybridization
TNBC: triple negative breast cancer
OS: overall survival
