# Supplementary material for: Expression of cancer-associated fibroblast related proteins in metastatic breast cancer: an immunohistochemical analysis
Source: J Transl Med. 2015 Jul 11;13:222. doi: 10.1186/s12967-015-0587-9 (PMC4702422; doi:10.1186/s12967-015-0587-9)
Supplement: Additional file 1: — Table S1. Source, clone, and dilution of the antibodies used. Table S2. Basal clinicopathologic characteristics of patients with metastatic breast cancer according to metastasis site. Table S3. Expression of CAF related proteins in the tumor cell compartment of metastatic breast cancer according to metastasis site.Table S4. Univariate analysis of the association between expression levels of CAF related proteins and overall survival by the log-rank test in metastatic breast cancer. [file 12967_2015_587_MOESM1_ESM.doc]

Supplementary Table 1. Source, clone, and dilution of the antibodies used in this study

| **Antibody** | **Company** | **Clone** | **Dilution** |
| --- | --- | --- | --- |
| *CAF phenotype related proteins* | |  |  |
| Podoplanin | Abcam, Cambridge, UK | 18H5 | 1:100 |
| Prolyl 4-hydroxylase | Abcam, Cambridge, UK | Polyclonal | 1:200 |
| FAPα | Abcam, Cambridge, UK | Polyclonal | 1:100 |
| S100A4 | Abcam, Cambridge, UK | Polyclonal | 1:100 |
| PDGFRα | Abcam, Cambridge, UK | Polyclonal | 1:100 |
| PDGFRβ | Abcam, Cambridge, UK | Y92 | 1:100 |
| NG2 | Abcam, Cambridge, UK | NG2 | 1:50 |
| *Molecular subtype related proteins* |  |  |  |
| ER | Thermo Scientific, San Diego, CA, USA | SP1 | 1:100 |
| PR | DAKO, Glostrup, Denmark | PgR | 1:50 |
| HER-2 | DAKO, Glostrup, Denmark | Polyclonal | 1:1500 |
| Ki-67 | Abcam, Cambridge, UK | MIB | 1:1000 |

| Supplementary Table 2. Basal clinicopathological characteristics of metastatic breast cancer according to the metastasis site | | | | | | |
| --- | --- | --- | --- | --- | --- | --- |
| Parameters | Total  N= 132 (%) | Bone  metastasis  n= 32 (%) | Brain  metastasis  n= 38 (%) | Liver  metastasis  n= 10 (%) | Lung  metastasis  n= 52 (%) | p-value |
| Age (years) |  |  |  |  |  | 0.097 |
| ≤50 | 68 (51.5) | 18 (56.2) | 17 (44.7) | 2 (20.0) | 31 (59.6) |  |
| >50 | 64 (48.5) | 14 (43.8) | 21 (55.3) | 8 (80.0) | 21 (40.4) |  |
| ER |  |  |  |  |  | **<0.001** |
| Negative | 63 (47.7) | 7 (21.9) | 25 (65.8) | 2 (20.0) | 29 (55.8) |  |
| Positive | 69 (52.3) | 25 (78.1) | 13 (34.2) | 8 (80.0) | 23 (44.2) |  |
| PR |  |  |  |  |  | **<0.001** |
| Negative | 91 (68.9) | 15 (46.9) | 37 (97.4) | 3 (30.0) | 36 (69.2) |  |
| Positive | 41 (31.1) | 17 (53.1) | 1 (2.6) | 7 (70.0) | 16 (30.8) |  |
| HER-2 |  |  |  |  |  | **0.047** |
| Negative | 89 (67.4) | 25 (78.1) | 19 (50.0) | 8 (80.0) | 37 (71.2) |  |
| Positive | 43 (32.6) | 7 (21.9) | 19 (50.0) | 2 (20.0) | 15 (28.8) |  |
| Molecular subtypes |  |  |  |  |  | **<0.001** |
| Luminal A | 47 (35.6) | 22 (68.8) | 4 (10.5) | 6 (60.0) | 15 (28.8) |  |
| Luminal B | 23 (17.4) | 4 (12.5) | 9 (23.7) | 2 (20.0) | 8 (15.4) |  |
| HER-2 | 27 (20.5) | 4 (12.5) | 12 (31.6) | 1 (10.0) | 10 (19.2) |  |
| TNBC | 35 (26.5) | 2 (6.2) | 13 (34.2) | 1 (10.0) | 19 (36.5) |  |
| Ki-67 LI (%) |  |  |  |  |  | **0.002** |
| ≤14 | 91 (68.9) | 29 (90.6) | 20 (52.6) | 9 (90.0) | 33 (63.5) |  |
| >14 | 41 (31.1) | 3 (9.4) | 18 (47.4) | 1 (10.0) | 19 (36.5) |  |
| Patients death | 44 (33.3) | 17 (53.1) | 11 (28.9) | 4 (40.0) | 12 (23.1) | **0.035** |

| Supplementary Table 3. Expression of cancer-associated fibroblast related proteins in metastatic breast cancer according to the metastasis site and molecular subtype | | | | | | | | | | | | | | | | | | | | |
| --- | --- | --- | --- | --- | --- | --- | --- | --- | --- | --- | --- | --- | --- | --- | --- | --- | --- | --- | --- | --- |
| Parameters | Bone metastasis, n= 32 (%) | | | | | Brain metastasis, n= 38 (%) | | | | | Liver metastasis, n= 10 (%) | | | | | Lung metastasis, n= 52 (%) | | | | |
|  | A | B | H | T | *P* | A | B | H | T | *P* | A | B | H | T | *P* | A | B | H | T | *P* |
| Podoplanin (T) |  |  |  |  | 0.556 |  |  |  |  | 0.452 |  |  |  |  | 0.302 |  |  |  |  | 0.700 |
| Negative | 16 (72.7) | 3 (75.0) | 4 (100.0) | 2 (100.0) |  | 3 (75.0) | 8 (88.9) | 12 (100.0) | 11 (84.6) |  | 5 (83.3) | 1 (50.0) | 0 (0.0) | 1 (100.0) |  | 10 (66.7) | 5 (62.5) | 6 (60.0) | 9 (47.4) |  |
| Positive | 6 (27.3) | 1 (25.0) | 0 (0.0) | 0 (0.0) |  | 1 (25.0) | 1 (11.1) | 0 (0.0) | 2 (15.4) |  | 1 (16.7) | 1 (50.0) | 1 (100.0) | 0 (0.0) |  | 5 (33.3) | 3 (37.5) | 4 (40.0) | 10 (52.6) |  |
| Podoplanin (S) |  |  |  |  | 0.118 |  |  |  |  | 0.496 |  |  |  |  | **0.019** |  |  |  |  | 0.139 |
| Negative | 17 (77.3) | 3 (75.0) | 2 (50.0) | 0 (0.0) |  | 4 (100.0) | 9 (100.0) | 10 (83.3) | 12 (92.3) |  | 6 (100.0) | 2 (100.0) | 0 (0.0) | 1 (100.0) |  | 13 (86.7) | 8 (100.0) | 10 (100.0) | 14 (73.7) |  |
| Positive | 5 (22.7) | 1 (25.0) | 2 (50.0) | 2 (100.0) |  | 0 (0.0) | 0 (0.0) | 2 (16.7) | 1 (7.7) |  | 0 (0.0) | 0 (0.0) | 1 (100.0) | 0 (0.0) |  | 2 (13.3) | 0 (0.0) | 0 (0.0) | 5 (26.3) |  |
| Prolyl 4-hydroxylase (T) |  |  |  |  | 0.833 |  |  |  |  | **0.003** |  |  |  |  | N/A |  |  |  |  | 0.607 |
| Negative | 7 (31.8) | 2 (50.0) | 1 (25.0) | 1 (50.0) |  | 1 (25.0) | 8 (88.9) | 1 (8.3) | 6 (46.2) |  | 6 (100.0) | 2 (100.0) | 1 (100.0) | 1 (100.0) |  | 1 (6.7) | 0 (0.0) | 1 (10.0) | 3 (15.8) |  |
| Positive | 15 (68.2) | 2 (50.0) | 3 (75.0) | 1 (50.0) |  | 3 (75.0) | 1 (11.1) | 11 (91.7) | 7 (53.8) |  | 0 (0.0) | 0 (0.0) | 0 (0.0) | 0 (0.0) |  | 14 (93.3) | 8 (100.0) | 9 (90.0) | 16 (84.2) |  |
| FAPα (T) |  |  |  |  | 0.621 |  |  |  |  | 0.086 |  |  |  |  | 0.864 |  |  |  |  | 0.473 |
| Negative | 20 (90.9) | 3 (75.0) | 4 (100.0) | 2 (100.0) |  | 4 (100.0) | 9 (100.0) | 8 (66.7) | 12 (92.3) |  | 5 (83.3) | 2 (100.0) | 1 (100.0) | 1 (100.0) |  | 14 (93.3) | 8 (100.0) | 10 (100.0) | 19 (100.0) |  |
| Positive | 2 (9.1) | 1 (25.0) | 0 (0.0) | 0 (0.0) |  | 0 (0.0) | 0 (0.0) | 4 (33.3) | 1 (7.7) |  | 1 (16.7) | 0 (0.0) | 0 (0.0) | 0 (0.0) |  | 1 (6.7) | 0 (0.0) | 0 (0.0) | 0 (0.0) |  |
| FAPα (S) |  |  |  |  | 0.926 |  |  |  |  | 0.288 |  |  |  |  | 0.644 |  |  |  |  | 0.915 |
| Negative | 21 (95.5) | 4 (100.0) | 4 (100.0) | 2 (100.0) |  | 4 (100.0) | 9 (100.0) | 11 (91.7) | 10 (76.9) |  | 5 (83.3) | 1 (50.0) | 1 (100.0) | 1 (100.0) |  | 14 (93.3) | 7 (87.5) | 9 (90.0) | 18 (94.7) |  |
| Positive | 1 (4.5) | 0 (0.0) | 0 (0.0) | 0 (0.0) |  | 0 (0.0) | 0 (0.0) | 1 (8.3) | 3 (23.1) |  | 1 (16.7) | 1 (50.0) | 0 (0.0) | 0 (0.0) |  | 1 (6.7) | 1 (12.5) | 1 (10.0) | 1 (5.3) |  |
| S100A4 (T) |  |  |  |  | 0.650 |  |  |  |  | **0.034** |  |  |  |  | **0.019** |  |  |  |  | 0.807 |
| Negative | 15 (68.2) | 3 (75.0) | 2 (50.0) | 2 (100.0) |  | 2 (50.0) | 9 (100.0) | 5 (41.7) | 6 (46.2) |  | 6 (100.0) | 0 (0.0) | 0 (0.0) | 1 (100.0) |  | 9 (60.0) | 4 (50.0) | 4 (40.0) | 10 (52.6) |  |
| Positive | 7 (31.8) | 1 (25.0) | 2 (50.0) | 0 (0.0) |  | 2 (50.0) | 0 (0.0) | 7 (58.3) | 7 (53.8) |  | 0 (0.0) | 2 (100.0) | 1 (100.0) | 0 (0.0) |  | 6 (40.0) | 4 (50.0) | 6 (60.0) | 9 (47.4) |  |
| S100A4 (S) |  |  |  |  | **0.015** |  |  |  |  | 0.578 |  |  |  |  | N/A |  |  |  |  | 0.527 |
| Negative | 17 (77.3) | 4 (100.0) | 1 (25.0) | 0 (0.0) |  | 4 (100.0) | 9 (100.0) | 12 (100.0) | 12 (92.3) |  | 6 (100.0) | 2 (100.0) | 1 (100.0) | 1 (100.0) |  | 13 (86.7) | 6 (75.0) | 9 (90.0) | 18 (94.7) |  |
| Positive | 5 (22.7) | 0 (0.0) | 3 (75.0) | 2 (100.0) |  | 0 (0.0) | 0 (0.0) | 0 (0.0) | 1 (7.7) |  | 0 (0.0) | 0 (0.0) | 0 (0.0) | 0 (0.0) |  | 2 (13.3) | 2 (25.0) | 1 (10.0) | 1 (5.3) |  |
| PDGFRα (T) |  |  |  |  | **0.037** |  |  |  |  | 0.131 |  |  |  |  | N/A |  |  |  |  | **0.034** |
| Negative | 18 (81.8) | 4 (100.0) | 1 (25.0) | 2 (100.0) |  | 2 (50.0) | 9 (100.0) | 7 (58.3) | 8 (61.5) |  | 6 (100.0) | 2 (100.0) | 1 (100.0) | 1 (100.0) |  | 15 (100.0) | 7 (87.5) | 6 (60.0) | 12 (63.2) |  |
| Positive | 4 (18.2) | 0 (0.0) | 3 (75.0) | 0 (0.0) |  | 2 (50.0) | 0 (0.0) | 5 (41.7) | 5 (38.5) |  | 0 (0.0) | 0 (0.0) | 0 (0.0) | 0 (0.0) |  | 0 (0.0) | 1 (12.5) | 4 (40.0) | 7 (36.8) |  |
| PDGFRα (S) |  |  |  |  | 0.254 |  |  |  |  | N/A |  |  |  |  | N/A |  |  |  |  | **0.042** |
| Negative | 18 (81.8) | 4 (100.0) | 2 (50.0) | 1 (50.0) |  | 4 (100.0) | 9 (100.0) | 12 (100.0) | 13 (100.0) |  | 6 (100.0) | 2 (100.0) | 1 (100.0) | 1 (100.0) |  | 11 (73.3) | 7 (87.5) | 10 (100.0) | 19 (100.0) |  |
| Positive | 4 (18.2) | 0 (0.0) | 2 (50.0) | 1 (50.0) |  | 0 (0.0) | 0 (0.0) | 0 (0.0) | 0 (0.0) |  | 0 (0.0) | 0 (0.0) | 0 (0.0) | 0 (0.0) |  | 4 (26.7) | 1 (12.5) | 0 (0.0) | 0 (0.0) |  |
| PDGFRβ (S) |  |  |  |  | 0.366 |  |  |  |  | 0.452 |  |  |  |  | 0.188 |  |  |  |  | 0.317 |
| Negative | 12 (54.5) | 4 (100.0) | 2 (50.0) | 1 (50.0) |  | 3 (75.0) | 8 (88.9) | 12 (100.0) | 11 (84.6) |  | 5 (83.3) | 2 (100.0) | 1 (100.0) | 0 (0.0) |  | 6 (40.0) | 2 (25.0) | 3 (30.0) | 11 (57.9) |  |
| Positive | 10 (45.5) | 0 (0.0) | 2 (50.0) | 1 (50.0) |  | 1 (25.0) | 1 (11.1) | 0 (0.0) | 2 (15.4) |  | 1 (16.7) | 0 (0.0) | 0 (0.0) | 1 (100.0) |  | 9 (60.0) | 6 (75.0) | 7 (70.0) | 8 (42.1) |  |
| NG2 (S) |  |  |  |  | N/A |  |  |  |  | 0.578 |  |  |  |  | N/A |  |  |  |  | 0.473 |
| Negative | 22 (100.0) | 4 (100.0) | 4 (100.0) | 2 (100.0) |  | 4 (100.0) | 9 (100.0) | 12 (100.0) | 12 (92.3) |  | 6 (100.0) | 2 (100.0) | 1 (100.0) | 1 (100.0) |  | 14 (93.3) | 8 (100.0) | 10 (100.0) | 19 (100.0) |  |
| Positive | 0 (0.0) | 0 (0.0) | 0 (0.0) | 0 (0.0) |  | 0 (0.0) | 0 (0.0) | 0 (0.0) | 1 (7.7) |  | 0 (0.0) | 0 (0.0) | 0 (0.0) | 0 (0.0) |  | 1 (6.7) | 0 (0.0) | 0 (0.0) | 0 (0.0) |  |
| T, tumor cell component, S, stromal component, A, luminal A, B, luminal B, H, HER-2 type, T, triple negative breast cancer | | | | | | | | | | | | | | | | | | | | |

Supplementary Table 4. Univariate analysis of cancer-associated fibroblast related protein expression and overall survival via the log-rank test

| Parameters | Total  N= 162 (%) | |
| --- | --- | --- |
|  | Mean survival  (95% CI) months | p-value |
| Podoplanin (T) |  | 0.074 |
| Negative | 123 (106-139) |  |
| Positive | 75 (58-92) |  |
| Podoplanin (S) |  | 0.307 |
| Negative | 117 (101-133) |  |
| Positive | 88 (49-128) |  |
| Prolyl 4-hydroxylase (T) |  | 0.641 |
| Negative | 117 (90-143) |  |
| Positive | 107 (89-126) |  |
| FAPα (T) |  | 0.920 |
| Negative | 114 (98-129) |  |
| Positive | 67 (51-83) |  |
| FAPα (S) |  | 0.923 |
| Negative | 114 (98-129) |  |
| Positive | 97 (48-145) |  |
| S100A4 (T) |  | 0.901 |
| Negative | 112 (94-130) |  |
| Positive | 108 (81-135) |  |
| S100A4 (S) |  | 0.935 |
| Negative | 112 (97-128) |  |
| Positive | 102 (72-133) |  |
| PDGFRα (T) |  | 0.718 |
| Negative | 114 (98-130) |  |
| Positive | 74 (60-89) |  |
| PDGFRα (S) |  | 0.889 |
| Negative | 113 (97-128) |  |
| Positive | 84 (54-114) |  |
| PDGFRβ (S) |  | 0.882 |
| Negative | 112 (94-130) |  |
| Positive | 114 (90-138) |  |
| NG2 (S) |  | N/A |
| Negative | N/A |  |
| Positive | N/A |  |
